# Splenic marginal zone B-cell lymphoma associated with ruptured breast implants: case report and review of the literature

**DOI:** 10.1080/23320885.2021.1891903

**Published:** 2021-04-05

**Authors:** Mark G. Evans, Melissa A. Mueller, Frederik Chen, Larry S. Nichter

**Affiliations:** a Department of Pathology and Laboratory Medicine, University of California, Irvine, CA, USA;; b Division of Plastic Surgery, Department of Surgery, University of Indiana, Indianapolis, IN, USA;; c Department of Radiology, Mayo Clinic, Phoenix, AZ, USA; d Department of Plastic Surgery, University of California, Irvine, CA, USA;; e Pacific Center for Plastic Surgery & Mission Plasticos, Newport Beach, CA, USA

**Keywords:** Breast implant, splenic marginal zone lymphoma, B-cell lymphoma, splenomegaly, augmentation mammoplasty

## Abstract

We describe splenomegaly and bilateral grade 2 Baker breast capsular contracture in a woman who had undergone augmentation mammoplasty. This case represents the first documented instance of splenic marginal zone lymphoma, and is among the rare reports of B-cell lymphoma, arising in a patient with breast implants.

## Introduction

The medical literature currently contains twelve cases of B-cell lymphoma occurring in patients with breast implants, including follicular lymphoma, primary cutaneous follicle center lymphoma, intravascular large-cell lymphoma, diffuse large B-cell lymphoma, marginal zone lymphoma, and plasmablastic lymphoma ([1–11], summarized in Table 1). An association between B-cell lymphomas and implants has been previously dismissed given the heterogeneity of such lymphoma cases. However, there are noteworthy commonalities amongst the cases. Many involved textured breast implants that had been compromised. The case presented here is the first case of splenic marginal zone B-cell lymphoma associated with breast implants. While it is unclear whether the ruptured implants directly contributed to the development of lymphoma, silicone granulomas from ruptured implants may have provided an immunologic stimulus. Plastic surgeons should be encouraged to report all cases of lymphoma associated with implants in order to further investigate this relationship.

**Table 1.** Cases of B-cell lymphomas associated with breast implants.

Study	Age (years)	Implant	Time from implantation to lymphoma (years)	Presentation	Treatment	Lymphoma Diagnosis
Cook et al. 1995	56	Silicone Polyurethane	6	Palpable mass and contracture	Mass resection, contracture correction, implant replacement	Follicular lymphoma
Nichter et al. 2012	58	Saline and silicone No additional information available	22	Contracture and axillary lymphadenopathy	Implant removal and capsulectomy	Follicular lymphoma and nodal marginal zone lymphoma
Jones et al. 2014	69	Silicone Smooth, moderate plus	6	Pain and swelling around sternum	Radiation therapy	Primary cutaneous follicle center lymphoma
Smith et al. 2014	83	Silicone Smooth Arion	44	Swelling and fluid surrounding right breast	Implant removal and complete capsulectomy	Diffuse large B-cell lymphoma, not otherwise specified
Moling et al. 2016	48	Silicone Polytech 44-G	4	Fever, splenomegaly, and hemophagocytic lymphohistiocytosis	Implant removal, partial capsulectomy, R-CHOP^a^, intrathecal methotrexate	Intravascular large B-cell lymphoma
Chen et al. 2018	34	Silicone Poly-implant prosthesis	14	Recurrent capsular contracture	Implant removal, mastectomy, complete capsulectomy, rituximab	Low-grade B-cell lymphoma/extranodal marginal zone lymphoma
Har-Shai et al. 2019	59	Silicone Textured NAGOR	11	Swelling and fluid surrounding left breast	Implant removal and complete capsulectomy	Anaplastic large cell lymphoma and extranodal marginal zone lymphoma
Geethakumari et al. 2019	74	Saline No additional information available	40	Pain, swelling, and fluid surrounding left breast	Implant removal, complete capsulectomy, V-EPOCH^b^ + bortezomib, consolidative radiation	Plasmablastic lymphoma
Larrimore et al. 2019	70	Silicone Nontextured	28	Asymptomatic breast mass on imaging	Implant removal and complete capsulectomy	Diffuse large B-cell lymphoma, not otherwise specified
Evans et al. 2020	69	Silicone Textured McGhan	11	Swelling and fluid surrounding right breast	Implant removal, complete capsulectomy, bendamustine + rituximab	Anaplastic large cell lymphoma and extranodal marginal zone lymphoma
Evans et al. 2020	62	Silicone Textured McGhan	36	Swelling and fluid surrounding right breast	Implant removal and complete capsulectomy	Extranodal marginal zone lymphoma
Evans et al. 2020	61	Saline No additional information available	21	Swelling and painful mass	R-CHOP^a^	Diffuse large B-cell lymphoma

## Case report

A 56-year-old woman presented with abdominal pain, bloating, early satiety, changes in bowel habits, and weight loss. Her past medical history included a ten-year history of progressive fatigue prompting early retirement at age 50 and a five-year history of left breast tenderness near her inframammary fold. She had undergone augmentation mammoplasty with subpectoral Dow Corning double lumen smooth implants 27 years previously and had never experienced a need for replacement.

**Figure 1.**
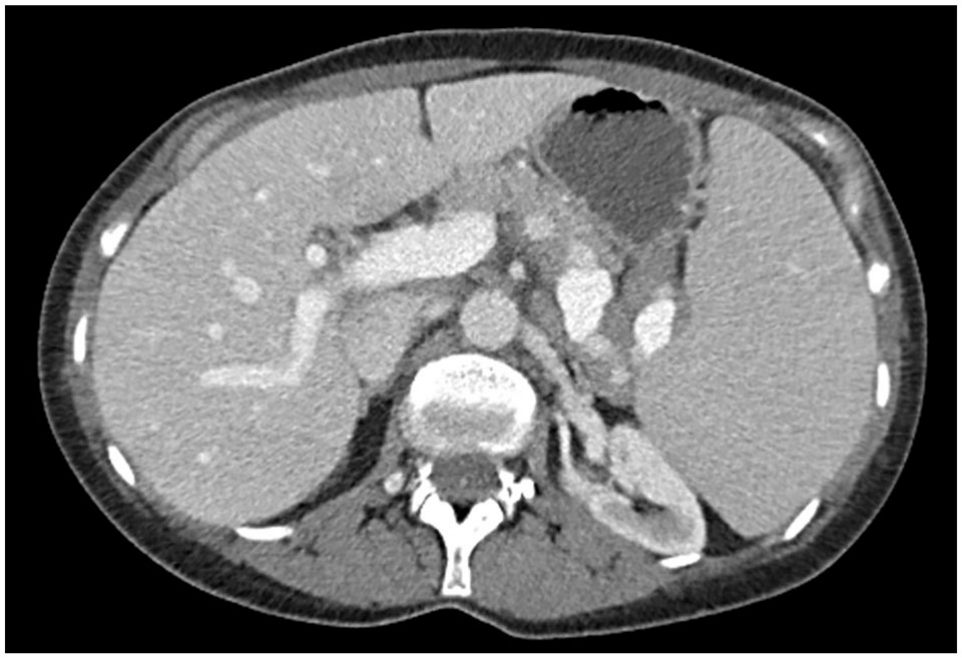
The patient’s abdominal CT scan demonstrates massive splenomegaly with adjacent abdominal lymphadenopathy.

**Figure 2.**
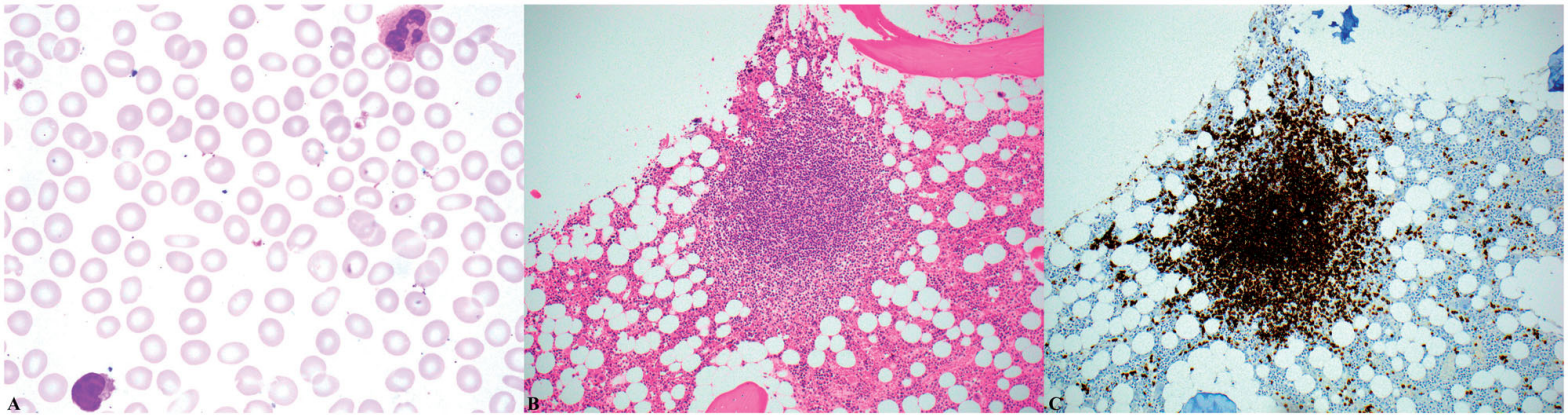
Microscopic examination of the patient’s peripheral blood smeared reveals atypical lymphocytes with villous morphology (A, 1000x magnification). Her bone marrow contained prominent lymphoid aggregates accounting for approximately 20% of the marrow’s cellularity (B, 200x magnification) that were composed of B-cells positive for CD20 by immunohistochemistry (C, 200x magnification).

An abdominal CT scan revealed splenomegaly, enlarged splenic hilar lymph nodes, and borderline-enlarged periaortic lymph nodes (Figure 1). Subsequent peripheral blood analysis and flow cytometric immunophenotyping, revealed pancytopenia and a clonal B-cell population with nonspecific immunophenotype and villous morphology. Her subsequent bone marrow aspirate and core biopsy were most consistent with splenic marginal zone lymphoma, occupying approximately 20% of marrow cellularity. Immunostains were positive for CD20 and negative for Cyclin D1 (Figure 2). Flow cytometry of the bone marrow showed a monoclonal B-cell population expressing CD20 (bright), CD45, FMC-7, CD19, CD11c (dim), CD22 (dim), and kappa immunoglobulin light chain restriction. The monoclonal B-cells were negative for CD5, CD10, and CD23.

A PET-CT scan demonstrated mildly hypermetabolic epigastric lymph nodes, hypermetabolic splenomegaly consistent with lymphoma, left breast increased metabolic activity suspicious for capsulitis, and bilateral findings suspicious for implant rupture. Subsequent breast MRI showed right implant intra-capsular contained rupture and left implant rupture with extracapsular silicone extension. There were two discrete 5-mm foci of extracapsular silicone along the inferior medial aspect of the left implant. Physical exam showed bilateral grade 2 Baker capsular contracture and no lymphadenopathy. The patient received weekly rituximab treatment for four weeks and also underwent bilateral capsulectomy and implant removal. Histologic examination of the excised capsular tissue demonstrated a marked inflammatory response and silicone granulomata, but no overt malignancy. On follow up eight years after therapy, the patient is currently alive without evidence of disease.

## Discussion

Splenic marginal zone B-cell lymphoma is a rare malignancy, accounting for less than 2% of all lymphoma cases [12]. It was first described in 1992 and is now considered a separate entity in the World Health Organization (WHO) classification [13]. The three types of marginal zone B-cell lymphomas are splenic marginal zone lymphoma (SMZL), nodal marginal zone lymphoma (NMZL), and extranodal marginal zone lymphoma of mucosa-associated lymphoid tissue (MALT lymphoma). The diagnosis of SMZL is made based on lymphocyte morphology, immunophenotype, cytogenetic abnormalities, bone marrow histology, and spleen histology if available. When microscopic examination of the spleen is not possible, clinical splenomegaly and typical morphologic and immunophenotypic blood and bone marrow findings are sufficient to make a diagnosis.

SMZL typically affects patients in the sixth decade without gender predominance. Main features include symptomatic massive splenomegaly, lymphocytosis, and cytopenias. Lymphadenopathy and extralymphatic organ involvement are uncommon [14]. Although no therapy is curative, this lymphoma type is indolent in nature. Median survival is roughly 10 years and there is greater than 60% 5-year survival [15–20].

An association may exist between marginal zone lymphomas and sustained immunologic stimulus. For example, gastric MALT lymphoma often arises in the setting of *Helicobacter pylori* infection. SMZL is associated with viruses, such as hepatitis C virus and Kaposi’s sarcoma- associated herpes virus [21–24]. One study found that autoimmune phenomena were present in 20% of SMZL patients [25]. In the case presented here, the patient had nodules near her left inframammary fold for five years, which were later found to be silicone granulomas from implant rupture. While it is unclear whether the ruptured implant directly contributed to the development of lymphoma, silicone granulomas from ruptured implants may have provided such an immunologic stimulus.

Of note, silicone prostheses have been reported in association with lymphoma arising at anatomic sites other than the breasts. To date, two cases of anaplastic large cell lymphoma have been documented adjacent to gluteal implants [26,27]. These patients lend support to the hypothesis that silicone leakage contributed to lymphoma development. While the argument can be made for loco-regional lymphomas occurring as a result of close-proximity immunologic stimuli, the data is less convincing as to whether disrupted implants are responsible for systemic lymphomas. The medical literature currently describes two patients with compromised silicone implants who presented with follicular lymphoma and intravascular large B-cell lymphoma outside of their breast tissue [3,4]. However, systemic lymphomas arising in women with breast implants could be purely coincidental. Upon conducting a Surveillance, Epidemiology, and End Results (SEER) incidence database query from 1975 to 2019, we have detected no statistically significant correlation between B-cell lymphoma development and the presence of breast implants. Similar findings have been discussed previously [28]. Therefore, we must be hesitant to infer causation between breast augmentation and B-cell lymphoma based on the less than 15 published cases.

## Conclusions

This case is the first report of splenic marginal zone lymphoma associated with ruptured silicone implants. There are now thirteen reports of B-cell lymphomas developing in implanted patients. Several involved leaking or ruptured silicone implants. Because of the rarity of B-cell lymphomas in augmented patients, there is less likely to be an association between B-cell lymphomas and ruptured silicone implants. To further investigate this possible association, plastic surgeons should report all cases of lymphoma, both B- and T-cell, in the context of breast implants.
